# Delivering primary health care as envisioned: A model of health and well-being guiding community-governed primary care organizations

**DOI:** 10.1108/JICA-02-2018-0014

**Published:** 2018-07-02

**Authors:** Jennifer Rayner, Laura Muldoon, Imaan Bayoumi, Dale McMurchy, Kate Mulligan, Wangari Tharao

**Affiliations:** 1 Centre for Studies in Family Medicine, The University of Western Ontario, Toronto, Canada; 2 Department of Research and Evaluation, Association of Ontario’s Health, Toronto, Canada; 3 Somerset West CHC, Ottawa, Canada; 4 Department of Family Medicine, Queen’s University, Kingston, Canada; 5 Dale McMurchy Consulting, Norland, Canada; 6 Department of Social and Behavioural Health Sciences, Dalla Lana School of Public Health, University of Toronto, Toronto, Canada; 7 Women’s Health in Women’s Hands, Toronto, Canada

**Keywords:** Health and well-being, Multi-disciplinary teamwork, Holistic approaches to care, Integrated health care, Primary health care

## Abstract

**Purpose:**

For over 40 years, Canadian and international bodies have endorsed comprehensive primary health care (PHC), yet very little work has been done to describe how services and programs are delivered within these organizations. Because health equity is now of greater interest to policy makers and the public, it is important to describe an evidence-informed framework for the delivery of integrated and equitable PHC. The purpose of this paper is to describe the development of a “Model of Health and Well-being” (MHWB) that provides a roadmap to the delivery of PHC in a successful network of community-governed PHC organizations in Ontario, Canada.

**Design/methodology/approach:**

The MHWB was developed through an iterative process that involved members of community-governed PHC organizations in Ontario and key stakeholders. This included literature review and consultation to ensure that the model was evidence informed and reflected actual practice.

**Findings:**

The MHWB has three guiding principles: highest quality health and well-being for people and communities; health equity and social justice; and community vitality and belonging. In addition, there are eight attributes that describe how services are provided. There is a reasonable evidence base underpinning the all principles and attributes.

**Originality/value:**

As comprehensive, equitable PHC organizations become increasingly recognized as critical parts of the health care system, it is important to have a means to describe their approach to care and the values that drive their care. The MHWB provides a blueprint for comprehensive PHC as delivered by over 100 Community Governed Primary Health Care (CGPHC) organizations in Ontario. All CGPHC organizations have endorsed, adopted and operationalized this model as a guide for optimum care delivery.

## Background

Ontario, Canada has a population of 14,193,384, spread across 1,076,395 square km. Population centers vary from Toronto, with over six million inhabitants, to small hamlets and remote communities. Health care is mostly publicly funded, and primary care is mostly delivered by groups and individual physicians running private practices which conform to different payment and delivery models. Barbara Starfield’s (1998) classic definition of PC describes most models for PC in Ontario: “that level of a health service system that provides entry into the system for all new needs and problems, provides person-focused (not disease-oriented) care over time, provides care for all but very uncommon or unusual conditions, and coordinates or integrates care provided elsewhere by others.” At the same time, most Ontario PC delivery models would not embody the broader concept of primary health care (PHC), which is an approach to care that includes services delivered to individuals and communities with a focus on health promotion, disease prevention, health equity, and community involvement (Muldoon *et al.*, 2010; Aggarwal and Hutchison, 2012). This focus on PC and not PHC exists despite many reports and position papers (Mable and Marriott, 2012; Hutchison *et al.*, 2011) extolling the virtues of the broader PHC approach. There is little to guide the work of policy makers looking beyond PC in order to ensure health equity through more comprehensive PHC.

Ontario’s PC/PHC landscape is complex due to a multiplicity of funding and delivery models (Table I). The complexity of the system and its heavy reliance on private practitioners who do not form part of a province-wide delivery system inhibit collective action, performance management, quality improvement, collaborative long-term planning, and collective data sharing.

The PHC approach puts a conception of people and communities first – rather than a model of funding or of service provision – and represents an innovation in thinking for Ontario’s health care system. By placing people at the center, this approach moves away from privileging service providers and can facilitate the people-centered health systems transformation envisioned under the provincial government’s “Patients First” agenda (Ministry of Health and Long-Term Care, 2017). It also addresses ongoing concerns, about the appropriate “mix” of funding models for PC in Ontario: a focus on PHC allows for innovative funding models to arise over time while keeping the focus on the role of PHC in advancing population health, health equity and individual and collective well-being (Office of the Auditor General of Ontario, 2017).

Despite Canada’s commitment to provide high-quality health care, health inequities remain a pressing concern. Of concern are the persistent health care inequities affecting marginalized populations (Brown *et al.*, 2012; Health Council of Canada, 2010). Paradoxically, those who have the greatest health and social complexities sometimes have the least access to care (Brown *et al.*, 2012; World Health Organization, 2008).

In Ontario, Community Health Centres (CHCs) and other community-governed PHC models (such as Aboriginal Health Access Centres (AHACs) some Community Family Health Teams (CFHTs) and some Nurse Practitioner Led Clinics (NPLCs)) address the equity gap by providing care to populations who experience barriers to accessing health care (AOHC, “Membership”). Ontario CHCs are community-governed interprofessional PHC organizations that have existed for over 40 years and serve over 600,000 people in 110 communities throughout Ontario (AOHC, “CHC fact sheet”). AHACs were established in the mid-1990s, followed by CFHTs and NPLCs in the early 2000s to fill gaps in service to certain communities and population groups. People who attend one of these organizations may receive interprofessional care from doctors, nurse practitioners, nurses, dietitians, social workers, and other kinds of clinical health providers. In addition to individual-based care, when funding is available, health promoters, community workers, and others respond to health problems triggered by social, environmental, or economic factors through services, community programs, and advocacy.

**Figure 1:**
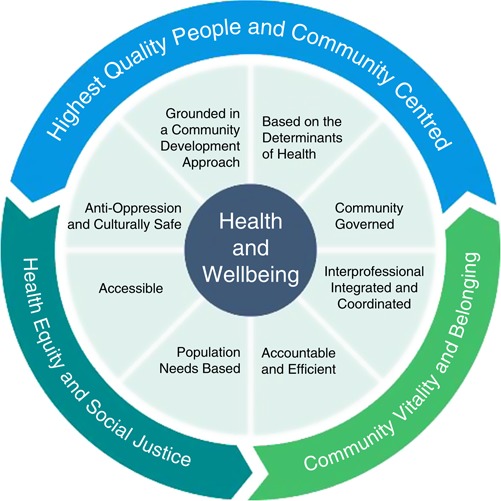
Model of Health and Well-being

In order to describe their approach to care provision and improve the quality of care, a group of these organizations developed a common evidence-informed roadmap, referred to as the Model of Health and Well-being (MHWB). This model is based on principles adapted from the World Health Organization and the 14 social determinants of health (underlying conditions that help determine a person’s health status, such as income, education, employment, food insecurity, housing social exclusion, gender, race, and disability). The MHWB contains eight attributes that taken together highlight the importance of an upstream, systemic perspective in the delivery of comprehensive PHC (see Figure 1). The model is based on the premise that people and communities who face barriers to health need access to integrated services that respond to the many different factors that have an impact on their health status. While the model originated in CHCs, the organizations that have endorsed and implemented the MHWB serve diverse populations based on the needs of their communities and include all Ontario Community Governed Primary Health Care (CGPHC) organizations (CHCs, AHACs, NPLCs, and CFHTs).

## Methods

The MHWB was created in 2013 by a team of sector leaders, health care practitioners, community developers, and health promoters. They started with the concept of people-centered health, from the World Health Organization (2009). The concept of the person became front and foremost and from there a series of values and attributes were included into a proposed model applicable at the organizational level. This iterative process was undertaken to confirm values, attributes, and definitions through a series of face-to-face meetings and interviews with stakeholders and clients.

Each version of the framework including definitions, attributes, and values was presented to sector leaders to ensure that the model was comprehensive and reflective of actual practice in the CGPHC organizations. Feedback was collated and revisions made to inform the next version of the model. This iterative process continued until the model was fully endorsed and adopted. A literature review was conducted to ensure that it was evidence informed.

### MHWB

The MHWB consists of three overarching goals: highest quality people and community-centered health and well-being; health equity and social justice; and community vitality and belonging. In addition to overarching goals, there are eight attributes that describe how services are provided. Definitions for the attributes are summarized below. Each attribute is supported by evidence underlining its importance to health and social care. Each attribute may be operationalized differently based on community need; however, specific service delivery has been provided as examples.

#### Based on the determinants of health

A common element in CGPHC organizations is the recognition of the influence of the DOH – upstream, non-clinical factors – on the health of the people they serve. There is an increasing body of evidence about what makes people healthy (Adler and Stewart, 2010; Marmot, 1999). These include key factors such as: income and social status; social support networks; education; employment/working conditions; social environments; physical environments; personal health practices and coping skills; healthy child development; biology and genetics; health services; gender; and culture (Public Health Agency of Canada, 2010). Each of these factors is important on its own, but they are also interrelated. There is growing social and biomedical evidence including relevant knowledge, documented associations, pathways and biological mechanisms to explain the interrelated impact of the DOH on health outcomes (Braveman *et al.*, 2011). There is also a growing body of literature demonstrating how the DOH operates at the individual and neighborhood levels (Macintyre *et al.*, 2008). Approximately 50 percent of population health outcomes in Canada are attributable to social and economic determinants which tend to cluster in particular communities (Keon and Pépin, 2009). In Ontario, there are many cases of avoidable illness and premature death because thousands of people simply cannot access the necessities to keep them healthy (Health Council of Canada, 2010).

Most CGPHC organizations have activities, advocacy and initiatives to mitigate the impact of poverty. In addition, childcare, transit, food boxes, and community gardens are found in many centers providing increased access to transportation and healthy food. Social isolation and increasing a sense of belonging is a priority across the CGPHC organizations and several have activities to bring people together.

#### Population needs based

The Public Health Agency of Canada defines the population health approach as “an approach to health that aims to improve the health of the entire population and to reduce health inequities among population groups”. This approach recognizes the importance of the DOH and focuses on the distribution of health across the populations. The population health approach recognizes the importance of intersectoral partnerships at the community level, across and among different levels of government and among health care providers and other professionals who have a role in influencing health (Dunn and Hayes, 1999; Neuwelt *et al.*, 2009). This is operationalized through partnerships between CGPHC and groups such as public health, libraries, shelters, home support services, youth programs, etc.

#### Anti-oppressive and culturally safe

Anti-oppressive practice includes the adoption of a set of non-discriminatory behaviors or skills, and an ongoing awareness of service providers’ own biases, judgments and potentially inequitable actions and their impact on the care that is provided (Larson, 2008). This reflective approach on the part of the provider is an important step toward minimizing the inequalities in care experienced by racialized groups. These practices are undertaken with the understanding that discrimination occurs in a variety of conscious and unconscious ways in everyday life. Oppression is reinforced not only by a series of overt actions but also by a range of subtle cues such as language choices and images. This approach is an important step toward mitigating unequal power relations that contribute to disparity in health outcomes.

Several researchers have suggested that indigenous people are often not provided health care within a culturally safe environment and experience high levels of systemic racism (Health Council of Canada, 2012; O’Sullivan, 2013; Shah and Reeves, 2012). Despite investments and efforts in health and socio-economic sectors within indigenous communities, current population studies reveal significant gaps in health outcomes compared to non-indigenous populations and high levels of systemic racism (Gracey and King, 2009; Smylie *et al.*, 2011; Allan and Smylie, 2015).

Compared to non-racialized Ontarians, racialized communities (both immigrants and Canadian born) face higher risk for particular health issues, including diabetes, heart disease, HIV/AIDs, and certain cancers (Hyman *et al.*, 2013; Nestel, 2012). Poor health is compounded by socio-economic barriers and inequities faced by many in racialized communities: poverty, precarious employment, social isolation/exclusion, and discrimination (Premji *et al.*, 2010; Smith and Mustard, 2009).

Attention to the DOH is critically important to address the health inequities faced by racialized communities and vulnerable immigrants/refugees. Newcomers are on average healthier than Canadian-born residents when they arrive but they lose this advantage over time (Setia *et al.*, 2012; Vang *et al.*, 2015). Compared to the overall Ontario population, immigrants, refugees, and racialized communities are less likely to access specialist care and mental health services (Hyman *et al.*, 2012).

Anti-oppressive practices are operationalized in organizations through attention to the spheres where discrimination manifests in everyday care. This emphasis on non-judgmental care delivery is also seen in the development of a harm reduction approach that is used with clients. This approach strives to meet clients “where they are” recognizing that all people have rights to care. This encourages providers to recognize that the human dignity of their client always presupposes the social barriers that bring them in for care. By taking this view, the client is transformed from the so-called “drug addict” to the person with a substance abuse disorder who requires a complex array of services. Recently, CHCs have housed the first supervised-injection sites in Ontario. Organizations seek to provide a culturally safe space so that diverse groups feel respected and engaged. This includes a reflection on the space itself such as images on posters, safe spaces, provision of food and childcare, and understanding individual learning styles. Staff training in anti-oppression and cultural safety is provided in many CGPHC. Various services are also provided by community health workers and peer workers delivering services.

#### Grounded in a community development approach

This attribute emphasizes that heath care is more than treating illness; it is about optimizing all the factors that allow people to live, learn, work, and play in their communities. Community capacity building has greater potential than clinical- or behavioral-based services to generate long-term sustainable improvements to the health of communities as a whole (Hawe, 2009).

Community development is the planned progression of all aspects of community well-being (economic, social, environmental, and cultural). It is a process whereby community members come together to take collective action and generate solutions to problems (Frank and Smith, 1999). The community development approach builds on community leadership, and the life experiences of community members to contribute to the health of their community. MHWB organizations work with communities to increase their capacity to improve community and individual health outcomes, and as a result their services and programs become more responsive to local community initiatives and needs. Examples are wide and varied and include projects such as community gardens and laundry co-ops. Pathways to Education is another example. This award-winning program was established at an urban CHC to provide support to students in an attempt to reduce the dropout rate. From 2001 to present, the dropout rate has been reduced from 56 to 10 percent; 95 percent of eligible high school youth have been enrolled in pathways and increased the university/college enrollment from 20 to 80 percent. An independent review suggested that the return on investment as from $25 to $1 (Boston Consulting Group, 2011).

#### Community-centered/community governance

MHWB organizations involve communities through a range of mechanisms, including focus groups, needs assessments, program planning, and board governance. A study by Church *et al.* (2006) suggested that CHCs provide a range of opportunities for “citizen participation” not seen in other parts of the health system leading to improvements in programs and services that better meet the needs of the community, increased community capacity, increased levels of trust in the community, and higher overall satisfaction.

Community-centered PHC systematically identifies and acts on community health needs using principles from epidemiology, PHC, preventive care, and health promotion (Longlett *et al.*, 2001) and stresses that the community context plays a role in the health of individuals (Muldoon *et al.*, 2010). Early evidence from as early as the 1940s showed that this model could have a substantial effect on the health of communities (Mullan and Epstein, 2002). Haggerty *et al.* have described community-centered PHC as existing almost exclusively in CHCs. Haggerty distinguishes between “community models” in which the populations served are defined by local geography vs the people who are served by “professional models” in which the populations served are the patients in the practice. The community-centered approach is described as being the most effective, providing the highest level of services and demonstrating the best possibility for controlling costs (Lamarche *et al.*, 2003).

All organizations that have adopted the MHWB are not for profit and governed by community boards. This provides a mechanism to be responsive to local needs and ensure representation and democratic ownership at the highest level of the organization. In addition, many organizations have community advisory councils that ensure more voices are heard at the leadership level. These councils provide ongoing feedback, raise concerns, priorities, and provide guidance for organizations.

#### Interprofessional, integrated, and coordinated

There is a robust body of evidence showing that interprofessional teams can improve health outcomes and access for people with chronic and complex conditions (Jacobson, 2012; Katon *et al.*, 2011). These benefits include significant improvements in health and wellness for people with chronic conditions and risk factors, compared with care provided by solo care providers (Dinh and Bounajm, 2013). Interprofessional teams develop care plans, address the medical and social needs of their patients, and provide better coordination of care (Goldman *et al.*, 2010). According to a study on the impact of PC teams on processes and outcomes of care, respondents who had access to an interprofessional team, particularly those with chronic conditions, were more likely to receive health promotion, disease prevention, and better coordination of care (Mable and Marriott, 2012).

All organizations that have adopted the MHWB work within collaborative interprofessional teams. They also seek to develop strong partnerships with external providers and community services. Often these organizations hire system navigators to identify and reduce barriers to care, diagnosis, and treatment. These staff identify, anticipate, and alleviate barriers to health and ensure that all internal and external services are coordinated.

#### Accountable and efficient

This model emphasizes accountability and efficiency as key attributes because in order to maximize resources and the services available, centers must emphasize continuous quality improvement and use resources effectively. All organizations are accountable to their communities, their funders and local health authorities. Each organization has accountability agreements with benchmarks and targets related to clinical care, service provision, and financial management. All CGPHC organizations participate in year quality improvement target setting and reporting. Most organizations are also accredited as well. The results of accreditation are public and validate that a commitment has been made to learning and improvement as well as demonstrated quality and appropriate risk management.

#### Accessible

Accessibility emphasizes access, equity, inclusiveness, and social justice. The MHWB stresses the importance of accessibility beyond usual working hours and includes ensuring access for people who encounter a diverse range of racial, cultural, linguistic, physical, social, economic, and geographic barriers which contribute to the risk of developing health problems. Removing barriers to accessibility includes the provision of culturally appropriate programs and services, programs for the non-insured, optimal service locations, and design of sites that are in compliance with accessibility legislation and offer extended hours and after-hours on-call services. Materials are written using plain language and are often translated into multiple languages. For example, in one urban area the client experience survey is offered in 17 different languages to ensure that all people can respond. Other examples include the provision of transportation (transit tickets, mobile units, volunteer driving programs). Often services are delivered in parks, schools, and shelters.

## Conclusion

There is ample evidence supporting each of the attributes included in the MHWB, especially for populations that experience barriers to care. The literature supports each attribute and while there are some overlaps, each of the attributes occupies a unique space. Each CGPHC organization has implemented the MHWB to reflect the needs of its population served and it was not difficult to find examples of how they have operationalized and implemented the model. Barriers to full implementation have primarily been funding.

PHC models that embody the upstream approaches and blend them with interprofessional care such as that of the MHWB has been shown to demonstrate positive outcomes. Studies include superior chronic disease management (Russell *et al.*, 2010), lower than expected emergency utilization (Glazier *et al.*, 2012), and higher than average cancer screening (Glazier and Rayner, 2015). There is less evidence describing how each attribute independently contributes to outcomes. Moreover, there is little evidence demonstrating how complex interventions addressing a number of the attributes act interdependently.

The MHWB is a living document and may be adapted in the future to ensure relevant to the changing world; however, the core principles and values will likely remain the same. Organizations that serve indigenous populations have refined this model to include cultural teachings and traditional practices. Future studies include in-depth case studies and ethnographic research to further understand the process of delivery and the impact of delivering comprehensive primary health care through this delivery model as well as summative evaluation to further examine overall impact of the model.

## Figures and Tables

**Table I:** Primary care models in Ontario (Health Force Ontario, 2017, family medicine compensation)

	Community Governed Primary Health Care (AHACs, CHCs, CFHTs, NPLCs)	Family Health Group (FHG)	Family Health Network (FHN)^a^	Family Health Organization (FHO)^a^
Physician reimbursement	Salary (CFHTs – blended salary)	Blended fee-for service	Blended capitation	Blended capitation
Governance	Community board (some NPLCs NP led)	Physician led	Physician led	Physician led
After-hours requirements	Yes	Yes	Yes	Yes
Accountability agreements with LHIN	CHCs only	No	No	No
Community outreach and health promotion	CHCs and AHACs	No	No	No
Interprofessional teams	Yes	No	Yes (FHTs only)	Yes (FHTs only)

**Note:** ^a^FHNs and FHOs may be part of a Family Health Team (FHT)

## References

[ref001] Adler N.E. and Stewart J. (2010), “Preface to the biology of disadvantage: socioeconomic status and health”, Annals of the New York Academy of Sciences, Vol. 1186 Nos 5-23, pp. 1-4. 20201864 10.1111/j.1749-6632.2009.05385.x

[ref201] Aggarwal M. and Hutchison B. (2012), Toward a Primary Care Strategy for Canada. Canadian Foundation for Healthcare Improvement, available at: www.cfhi-fcass.ca/Libraries/Reports/Primary-Care-Strategy-EN.sflb.ashx (accessed April 18, 2018).

[ref002] Allan B. and Smylie J. (2015), First Peoples, Second Class Treatment: The Role of Racism in the Health and Well-Being of Indigenous Peoples in Canada, The Wellesley Institute, Toronto, ON.

[ref003] Boston Consulting Group (2011), “BCG assessment of pathways to education”, available at: www.pathwaystoeducation.ca/sites/default/files/editor_uploads/pdf/BCG%20Assessment%20of%20Pathways.pdf (accessed March 7, 2018).

[ref004] Braveman P., Egerter S. and Williams D.R. (2011), “The social determinants of health: coming of age”, Annual Review of Public Health, Vol. 32, pp. 381-398, available at: 10.1146/annurev-publhealth-031210-101218 21091195

[ref202] Brown A.J., Varcoe C.M., Wong S.T., Smye V.L., Lavoie J., Littlejohn D., Tu D., Godwin O., Kraues M., Khan K.B., Fridkin A., Rodney P., O’Neil J. and Lennox S. (2012), “Closing the health equity gap: evidence-based strategies for primary health care organizations”, International Journal for Equity in Health, pp. 11-59. 23061433 10.1186/1475-9276-11-59 PMC3570279

[ref005] Church J., Triska O., Rondeau K., Wilson D., Wagner P.S., McKim B., Lafleur R., Gibbons K., Marko J., Chapman M., Mawji A. and Lafleur D. (2006), “Citizenship participation partnership project”, Technical Report No. 06-001, Centre for Health Promotion Studies, School of Public Health, University of Alberta, Edmonton.

[ref006] Dinh T. and Bounajm F. (2013), “Improving primary health care through collaboration: briefing 3 – measuring the missed opportunity”, The Conference Board of Canada, available at: www.conferenceboard.ca/e-library/abstract.aspx?did=5479 (accessed April 18, 2018).

[ref047] Dunn J.R. and Hayes M.V. (1999), “Toward a lexicon of population health”, Canadian Journal of Public Health/Revue Canadienne de Santé Publique, Vol. 90 No. 1, pp. S7-S10. 10686751 10.1007/BF03403570 PMC6979857

[ref048] Frank F. and Smith A. (1999), The Community Development Handbook: A Tool to Build Community Capacity, Human Resources Development Canada (HRDC), Quebec.

[ref007] Glazier R.H. and Rayner J. (2015), Examining Community Health Centres According to Geography and Priority Populations Served, 2011/12-2012/13: An ICES Chartbook, Institute for Clinical and Evaluative Sciences, Toronto, available at: https://healthcouncilcanada.ca/files/2.40.1-HealthPromo_appendicesDec2010.pdf (accessed April 18, 2018).

[ref008] Glazier R.H., Zagorski B.M. and Rayner J. (2012), Comparison of Primary Care Models in Ontario by Demographics, Case Mix and Emergency Department Use, 2008/09 to 2009/10, Institute for Clinical and Evaluative Sciences, Toronto, available at: https://healthcouncilcanada.ca/files/Aboriginal_Report_EN_web_final.pdf (accessed April 18, 2018).

[ref009] Goldman J., Meuser J., Rogers J., Lawrie L. and Reeves S. (2010), “Interprofessional collaboration in family health teams: an Ontario-based study”, Canadian Family Physician, Vol. 56 No. 10, pp. e368-e374. 20944025 PMC2954101

[ref010] Gracey M. and King M. (2009), “Indigenous health part 1: determinants and disease patterns”, Lancet, Vol. 374 No. 9683, pp. 65-75. 19577695 10.1016/S0140-6736(09)60914-4

[ref011] Hawe P. (2009), “The social determinants of health: how can a radical agenda be mainstreamed?”, Canadian Journal of Public Health, Vol. 100 No. 4, pp. 291-293. 19722343 10.1007/BF03403949 PMC6973944

[ref012] Health Council of Canada (2010), Stepping It Up: Moving the Focus From Health Care in Canada to a Healthier Canada, Toronto.

[ref013] Health Council of Canada (2012), Empathy, Dignity and Respect: Creating Cultural Safety for Aboriginal People in Urban Health Care, Toronto.

[ref014] Health Force Ontario (2017), “Family medicine compensation and practice models in Ontario”, available at: www.healthforceontario.ca/UserFiles/file/PracticeOntario/FM%20Compensation%20Practice%20Models%20EN.pdf (accessed March 12, 2017).

[ref015] Hutchison B., Levesque J., Strumph E. and Coyle N. (2011), “Primary health care in Canada: systems in motion”, The Milbank Quarterly, Vol. 89 No. 2, pp. 256-288. 21676023 10.1111/j.1468-0009.2011.00628.x PMC3142339

[ref017] Hyman I., Gucciardi E., Patychuk D., Rummens J.A., Shakya Y., Kljujic D., Bhamani M. and Boqaileh F. (2012), “Self-management, health service use and information seeking for diabetes care among black Caribbean immigrants in Toronto”, Canadian Journal of Diabetes, Vol. 38 No. 1, pp. 32-37. 10.1016/j.jcjd.2013.08.267 24485211

[ref018] Hyman I. and Wray R., and Toronto Public Health (2013), Health Inequalities and Racialized Groups: A Review of the Evidence, Toronto Public Health, Toronto.

[ref019] Jacobson P. (2012), Evidence Synthesis for the Effectiveness of Interprofessional Teams in Primary Care, Canadian Health Services Research Foundation, Ottawa.

[ref050] Katon W., Lin E., Von Korff M., Ciechanowsk I.P., Ludman E., Young B., Peterson D., Rutter C.M., McGregor M. and McCulloch D. (2011), “Collaborative care for depression and chronic illnesses”, New England Journal of Medicine, Vol. 364 No. 13, pp. 1278-1279.21449795

[ref020] Keon W. and Pépin L. (2009), A Healthy, Productive Canada: A Determinant of Health Approach, The Standing Senate Committee on Social Affairs, Science and Technology, Ottawa.

[ref021] Lamarche P., Beaulieu M., Pineault R., Contandriopoulos A., Denis J.L. and Haggerty J. (2003), Choices for Change: The Path for Restructuring Primary Healthcare Services in Canada, Canadian Health Services Research Foundation, Ottawa.

[ref022] Larson G. (2008), “Anti-oppressive practice in mental health”, Journal of Progressive Human Services, Vol. 19 No. 1, pp. 39-54.

[ref023] Longlett S.K., Kruse J.E. and Wesley R.M. (2001), “Community-oriented primary care: historical perspective”, The Journal of the American Board of Family Practice, Vol. 14 No. 1, pp. 54-63. 11206694

[ref025] Mable A. and Marriott J. (2012), Canadian Primary Healthcare Policy: The Evolving Status of Reform, Canadian Foundation for Healthcare Improvement Ottawa, Ontario.

[ref026] Macintyre S., Macdonald L. and Ellaway A. (2008), “Do poorer people have poorer access to local resources and facilities? The distribution of local resources by area deprivation in Glasgow, Scotland”, Social Science and Medicine, Vol. 67 No. 6, pp. 900-914. 18599170 10.1016/j.socscimed.2008.05.029 PMC2570170

[ref027] Marmot M. (1999), “Epidemiology of socioeconomic status and health: are determinants within countries the same as between countries?”, Annals of the New York Academy of Sciences, Vol. 896 No. 1, pp. 19-26. 10.1111/j.1749-6632.1999.tb08102.x 10681885

[ref028] Ministry of Health and Long-Term Care (2017), “Patients first action plan”, available at: www.health.gov.on.ca/en/ms/ecfa/healthy_change/ (accessed February 17, 2018).

[ref029] Muldoon L., Dahrouge S., Hogg W., Geneau R., Russell G. and Shortt M. (2010), “Community orientation in primary care practices: results from the comparison of models of primary health care in Ontario study”, Canadian Family Physician, Vol. 56 No. 7, pp. 676-683. 20631283 PMC2922817

[ref030] Mullan F. and Epstein L. (2002), “Community-oriented primary care: new relevance in a changing world”, American Journal of Public Health, Vol. 92 No. 11, pp. 1748-1755. 12406800 10.2105/ajph.92.11.1748 PMC3221479

[ref031] Nestel S. (2012), Colour Coded Health Care: The Impact of Race and Racism on Canadians’ Health, Wellesley Institute, Toronto.

[ref032] Neuwelt P., Mathesome D., Arrol B., Dowell A., Winnard D., Crampton P., Sheridan N. and Cumming J. (2009), “Putting population health into practice through primary health care”, The New Zealand Medical Journal, Vol. 122 No. 1290, pp. 98-104. 19319172

[ref033] O’Sullivan B. (2013), “Considering culture in Aboriginal care”, Canadian Medical Association Journal, Vol. 185 No. 1, pp. E27-E28. 23251019 10.1503/cmaj.109-4376 PMC3537803

[ref034] Office of the Auditor General of Ontario (2017), “Annual report”, Chapter 3, Section 3.03, Community Health Centres, Toronto.

[ref203] Premji S., Duguay P., Messing K. and Lippel K. (2010), “Are immigrants, ethnic and linguistic minorities over-represented in jobs with a high level of compensated risk? Results from a montreal, Canada study using census and workers’ compensation data”, American Journal of Industrial Medicine, Vol. 53, pp. 875-885. 20698020 10.1002/ajim.20845

[ref035] Public Health Agency of Canada (2010), “What makes Canadians healthy?”, available at: www.phac-aspc.gc.ca/ph-sp/determinants/determinants-eng.php♯unhealthy (accessed February 2, 2018).

[ref036] Russell G., Dahrouge S., Tuna M., Hogg W., Geneau R. and Gebremichael G. (2010), “Getting it all done: organizational factors linked with comprehensive primary care”, Family Practice, Vol. 27 No. 5, pp. 535-541. 20534791 10.1093/fampra/cmq037

[ref051] Setia M.S., Quesnel-Vallee A., Abrahamowicz M., Tousignant P. and Lynch J. (2012), “Different outcomes for different health measures in immigrants: evidence from a longitudinal analysis of the national population health survey (1994-2006)”, Journal of Immigrant and Minority Health, Vol. 14 No. 1, pp. 156-165. 21042935 10.1007/s10903-010-9408-7

[ref037] Shah C.P. and Reeves A. (2012), “Increasing aboriginal cultural safety among health care practitioners”, Canadian Journal of Public Health, Vol. 103 No. 5, p. e397. 23617997 10.1007/BF03404450 PMC6973920

[ref204] Smith P.M. and Mustard C.A. (2009), “Comparing the risk of work-related injuries between immigrants to Canada and Canadian-born labour market participants”, Occupational and Environmental Medicine, Vol. 66 No. 6, pp. 361-367. 18614627 10.1136/oem.2007.038646

[ref038] Smylie J., Prince C. and Mayo S. (2011), Our Health Counts, Hamilton, available at: www.ourhealthcounts.ca/images/PDF/OHC-Report-Hamilton-ON.pdf (accessed April 18, 2018).

[ref039] Starfield B. (1998), Primary Care: Balancing Health Needs, Services and Technology, 2nd ed., Oxford University Press, New York, NY and Oxford, pp. 8-9.

[ref040] Vang Z., Sigouin J., Flenon A. and Gagnon A. (2015), “The healthy immigrant effect in Canada: a systematic review”, Population Change and Lifecourse Strategic Knowledge Cluster Discussion Paper Series/Un Réseau stratégique de connaissances Changements de population et parcours de vie Document de travail, Vol. 3 No. 1, Article No. 4, available at: http://ir.lib.uwo.ca/pclc/vol3/iss1/4

[ref041] World Health Organization (2008), “World Health Report 2008 – primary health care (now more than ever)”, World Health Organization (WHO), Geneva, available at: www.who.int/whr/2008/en/ (accessed April 18, 2018).

[ref042] World Health Organization (2009), “Primary health care, including health system strengthening. Agenda item 12.4”, Sixty-Second World Health Assembly, Geneva, available at: www.who.int/hrh/resources/A62_12_EN.pdf (accessed April 18, 2018).

